# Afferent Fiber Remodeling in the Somatosensory Thalamus of Mice as a Neural Basis of Somatotopic Reorganization in the Brain and Ectopic Mechanical Hypersensitivity after Peripheral Sensory Nerve Injury

**DOI:** 10.1523/ENEURO.0345-16.2017

**Published:** 2017-04-03

**Authors:** Yuichi Takeuchi, Hironobu Osaki, Yuki Yagasaki, Yoko Katayama, Mariko Miyata

**Affiliations:** 1 Department of Physiology, School of Medicine, Tokyo Women’s Medical University, Tokyo 162-8666, Japan; 2 Division of Women Health Care Professionals and Researchers Support, TWMU Career Development Center for Medical Professionals, Tokyo Women’s Medical University, Tokyo 162-8666, Japan

**Keywords:** nerve injury, thalamus

## Abstract

Plastic changes in the CNS in response to peripheral sensory nerve injury are a series of complex processes, ranging from local circuit remodeling to somatotopic reorganization. However, the link between circuit remodeling and somatotopic reorganization remains unclear. We have previously reported that transection of the primary whisker sensory nerve causes the abnormal rewiring of lemniscal fibers (sensory afferents) on a neuron in the mouse whisker sensory thalamus (V2 VPM). In the present study, using transgenic mice whose lemniscal fibers originate from the whisker sensory principle trigeminal nucleus (PrV2) are specifically labeled, we identified that the transection induced retraction of PrV2-originating lemniscal fibers and invasion of those not originating from PrV2 in the V2 VPM. This anatomical remodeling with somatotopic reorganization was highly correlated with the rewiring of lemniscal fibers. Origins of the non-PrV2-origin lemniscal fibers in the V2 VPM included the mandibular subregion of trigeminal nuclei and the dorsal column nuclei (DCNs), which normally represent body parts other than whiskers. The transection also resulted in ectopic receptive fields of V2 VPM neurons and extraterritorial pain behavior on the uninjured mandibular region of the face. The anatomical remodeling, emergence of ectopic receptive fields, and extraterritorial pain behavior all concomitantly developed within a week and lasted more than three months after the transection. Our findings, thus, indicate a strong linkage between these plastic changes after peripheral sensory nerve injury, which may provide a neural circuit basis underlying large-scale reorganization of somatotopic representation and abnormal ectopic sensations.

## Significance Statement

Peripheral sensory nerve injury causes various plastic changes in the somatosensory pathway. However, the link between these plastic changes remains poorly understood. In the present study, taking advantage of a transgenic mouse line, whose somatotopic information on afferent fibers is specifically visualized, we identified that afferent fiber remodeling in the thalamus after sensory nerve injury mediates large-scale somatotopic reorganization. Since the afferent fiber remodeling in the thalamus concomitantly occurred and lasted along with reorganization of somatotopic representation in the thalamus and ectopic pain behavior, the afferent fiber remodeling with somatotopic reorganization in the thalamus could potentially be a neural basis of clinically problematic ectopic sensations.

## Introduction

Neural circuitry in the CNS is capable of remodeling in response to peripheral sensory nerve injury (Woolf and Mannion, 1999; Navarro et al., 2007; Kaas et al., 2008). At the neural circuit level, peripheral sensory nerve injury such as afferent fiber transection and ligation causes anatomic remodeling of presynaptic axon fibers throughout the sensory afferent pathway from the spinal cord to the cortex (Darian-Smith and Gilbert, 1994; Sengelaub et al., 1997; Florence et al., 1998; Jain et al., 2000; Okamoto et al., 2001; Graziano and Jones, 2009; Yamahachi et al., 2009). Peripheral sensory nerve injury also induces rapid remodeling of postsynaptic dendritic spines (Kim and Nabekura, 2011). Moreover, the efficacy of functional synaptic transmission is altered after injury at all levels along the afferent axis (Kohno et al., 2003; Takeuchi et al., 2012; Yu et al., 2012). From a systems perspective, peripheral sensory nerve injury causes reorganization of somatotopic representation of the brain, such as changes in size of receptive fields in the somatosensory axis in anesthetized animals (Merzenich et al., 1984; Waite, 1984; Garraghty and Kaas, 1991). Such reorganization of receptive fields can be large in scale and extends beyond the somatotopic border (Pons et al., 1991; Jones and Pons, 1998). Furthermore, peripheral sensory nerve injury also induces abnormal ectopic sensations in humans such as extraterritorial orofacial pain (Kobayashi et al., 2011; Renton et al., 2012; Tseng et al., 2012; Okada-Ogawa et al., 2015) and phantom referred sensation (e.g., hand sensations evoked by face touch), which occasionally results in pain (Ramachandran and Hirstein, 1998; Flor et al., 2006). Large-scale somatotopic reorganization also correlates with phantom pain sensation, suggesting a possible causal relationship (Flor et al., 1995; Grüsser et al., 2001). However, while there are scattered pieces of evidence from the neural circuit level to the systems level, the linkage between particular neural circuitry remodeling and the somatotopic reorganization that would ultimately lead to abnormal ectopic sensation remains unclear. This is mainly because the somatotopic information that remodeled axon fibers carry has not been fully identified.

The somatosensory thalamus receives lemniscal fibers (sensory afferents) from the brainstem and projects to the primary somatosensory cortex (Watson et al., 2012). Connections between lemniscal fibers and thalamic neurons in the whisker sensory thalamus (V2 VPM) of the mouse have been a representative model of functional circuit remodeling such as developmental synapse elimination (Arsenault and Zhang, 2006); innervation of a single lemniscal fiber to a mature V2 VPM neuron is completed via developmental synapse elimination (Arsenault and Zhang, 2006; Takeuchi et al., 2014). We have previously demonstrated that complete transection of the primary whisker sensory nerve of mice causes robust abnormal rewiring of lemniscal fibers, in that multiple lemniscal fibers are newly recruited onto a V2 VPM neuron [infraorbital nerve cut (IONC) model; Takeuchi et al., 2012]. Together with evidence accumulated in *in vivo* studies, we hypothesized that newly recruited lemniscal fibers originate from different sources, other than the whisker sensory principle trigeminal nucleus (PrV2), which leads to somatotopic reorganization and finally abnormal ectopic sensations.

In the present study, taking advantage of the IONC model and Krox20-Ai14, a transgenic mouse line, whose PrV2-origin lemniscal fibers are specifically visualized, we confirmed that newly recruited lemniscal fibers found in the IONC model originated specific brainstem nuclei, which normally represent the mandibular (V3) region of the face and/or other body parts. We also observed ectopic receptive fields of V2 VPM neurons and extraterritorial mechanical hypersensitivity in the corresponding V3 region. Furthermore, all these plastic changes emerged and lasted over a similar time course. Our results, thus, indicate that there is a close link between neural circuitry remodeling with somatotopic reorganization and ectopic sensations. We propose that the afferent remodeling in the thalamus after peripheral sensory nerve injury may serve as a neural basis for abnormal ectopic sensations such as extraterritorial orofacial pain and phantom referred sensation.

## Materials and Methods

### Animals

All experiments were approved by the Animal Care and Use Committee of the Tokyo Women’s Medical University and performed according to the institutional guidelines. To visualize the PrV2-origin lemniscal fibers, *Krox-20^cre^* mice (RRID:IMSR_JAX:025744; Voiculescu et al., 2000) and Cre-dependent tdTomato reporter mice (RRID:IMSR_JAX:007914; Madisen et al., 2010) were crossed, and the Cre-positive pups (Krox20-Ai14 mice) were used as previously described (Takeuchi et al., 2014). Wild-type C57BL/6 mice (RRID:IMSR_JAX:000664) were purchased from SLC. Mice were fed a commercial diet and water *ad libitum* with controlled temperature, humidity, and lighting (12/12 h light/dark cycle). Both sexes of postnatal day 21 (P21) adult mice were used. Numbers of mice used were 120 Krox20-Ai14 and 186 wild type.

### Genotyping

*Krox-20^cre^* mice were genotyped by a standard PCR procedure, using the following primers: CW-Cre2, 5’-ACC TGA TGG ACA TGT TCA GGG ATC G-3’ and CW-Cre3, 5’-TCC GGT TAT TCA ACT TGC ACC ATG C-3’, producing a 108-bp fragment from the *cre* allele (Iwasato et al., 2004). Krox20-Ai14 pups were genotyped by PCR and/or detecting tdTomato signals in vibrissal follicles expressed from late pregnancy (Voiculescu et al., 2000). Results of both typing methods were always consistent.

### IONC

The primary whisker sensory nerve (infraorbital nerve, ION) of the left side was exposed and completely transected under ketamine/xylazine anesthesia (80/10 mg/kg, i.p.) as previously described (Takeuchi et al., 2012). The cut was conducted on P21 or two-month-old mice. For long survival case after the cut, the ION was tightly ligated by 6-0 silken thread before cut, and a plate of ethylene-vinyl acetate copolymer was placed between stumps to prevent nerve regeneration (Kakizawa et al., 2005).

### Whisker deprivation

All mystacial vibrissae on the left side of the snout were deprived every other day from P21 to the perfusion day ranging from P28 to P32. Under a dissecting microscope, vibrissae of isoflurane-anesthetized mice were carefully plucked out using fine tweezers by applying slow and steady tension to the base of the vibrissa until the vibrissa slipped out of the follicle (Arsenault and Zhang, 2006).

### Histology

#### Perfusion and cryosectioning

Mice were deeply anesthetized with pentobarbital (i.p.) and transcardially perfused with ice-cold saline followed by 4% paraformaldehyde (PFA) and 0.2% picric acid in 0.1 M phosphate buffer (PB), pH 7.2–7.3. After removal, brains were postfixed overnight, infiltrated with sucrose gradient, blocked, and frozen on dry ice. Thalamic or brainstem sections (20-µm thickness, coronal or horizontal plane) were made using a freezing microtome. Sections were collected in antifreeze solution, rinsed in 0.1 M PBS, and then subjected to one of the staining procedures described below. All staining procedures were performed at room temperature unless otherwise noted. For series of quantitative fluorescence measurements (tdTomato fluorescence measurements, VGluT2 and VGluT2-MAP2 immunohistochemistry), eight consecutive horizontal sections, total 160-µm-thick, centered at the cloud of the tdTomato-labeled PrV2-origin lemniscal fiber terminals in the V2 VPM were collected from each Krox20-Ai14 mouse. Four alternating sections of them were used for fluorescent staining and measurements. The remaining four sections were subjected to cytochrome oxidase (CO) staining (Takeuchi et al., 2014).

#### Fluorescent Nissl counterstaining of cryosections

Sections were first mounted on glass slides. Sections were then incubated with PBS containing 0.3% Triton X-100 (PBS-X) for 10 min, washed with PBS, incubated with a fluoro-Nissl solution (N-21479 or N-21480; Thermo Fisher Scientific) for 40 min, washed with PBS, incubated with PBS-X for 10 min, and washed with PBS again. Sections were finally coverslipped with 50% glycerol and 2.5% triethylene diamine in PBS.

#### VGluT2 immunohistochemistry

All incubations were followed by washing with PBS-X. Sections were incubated successively with 10% normal goat serum (NGS) in PBS-X for 30 min, 1 µg/ml guinea pig anti-vesicular glutamate transporter type 2 (VGluT2) polyclonal antibody (Frontier Institute catalog #VGluT2-GP, RRID:AB_2571621; Miyazaki et al., 2003) in PBS-X containing 1% NGS and 0.02% sodium azide (PBS-XG) overnight, and Alexa Fluor 633-conjugated goat anti-guinea pig IgG (A-21105; Thermo Fisher Scientific) for 2 h. Sections were then mounted, counterstained, and coverslipped.

#### MAP2 and VGluT2 double immunohistochemistry

To examine contacts between V2 VPM neurons and lemniscal fiber terminals, they were simultaneously labeled with MAP2 and VGluT2, respectively. Sections were incubated successively with 10% normal donkey serum (NDS) in PBS-X for 30 min, a mixture of 1st antibodies (Frontier Institute catalog #MAP2-Go, RRID:AB_2571793 and Frontier Institute catalog #VGluT2-Rb, RRID:AB_2571619, 1 µg/ml for each) in PBS containing 1% NDS and 0.02% sodium azide (PBS-XD; Miura et al., 2006) overnight, and a mixture of 2nd antibodies (Alexa Fluor 647-conjugated donkey anti-goat IgG: A-21447 and Alexa Fluor 488-conjugated donkey anti-rabbit IgG: A-21206; Thermo Fisher Scientific) for 2 h. Sections were then mounted, counterstained, and coverslipped.

#### Anterograde labeling of the PrV2-origin lemniscal fibers

Four days after IONC, mice were anesthetized with a ketamine/xylazine cocktail (80/10 mg/kg, i.p.) and then mounted on a stereotaxic apparatus. An anterograde tracer, 2% biotinylated dextran amine (BDA; Thermo Fisher Scientific catalog #D1956, RRID:AB_2307337) was filled in a glass electrode (impedance: 0.2–5 MΩ; tip 2–5 µm). To inject BDA in the PrV2, the electrode was inserted 25° obliquely from the left occipital cortex (1.75–1.80 mm posterior and 1.70–1.75 mm lateral from the bregma) and then caudally advanced 5.70–5.90 mm (Franklin and Paxinos, 2008). To precisely locate the electrode in the PrV2, we monitored neural firing extracellularly along the track in response to tactile stimuli on the face with amplifiers (MEZ-8300 and AVH-11; Nihon Kohden). In the PrV2, BDA was iontophoretically ejected as anodal currents (+1–5 µA, 3 s on/off half duty cycle, 30 min). After 4 d of survival, mice were transcardially perfused with 4% PFA and 0.5% glutaraldehyde in 0.1 M PB. After cryoprotection, the brains were horizontally sectioned at 60-µm-thick and collected serially. Sections were then subjected to CO staining, standard ABC-amplification, peroxidase/benzidine reaction with nickel enhancement, mounted, dehydrated, cleared, and coverslipped (Veinante and Deschênes, 1999). Individual lemniscal fibers were three-dimensionally reconstructed using a computer-assisted microscope system (Eclipse E800; Nikon) with a 40× PlanApo objective lens (40×/0.95; Nikon) and mapping program (Neurolucida, RRID:SCR_001775; Honda et al., 2012). Reconstructed data were quantitatively analyzed with Neurolucida Explore software and IGOR Pro software (RRID:SCR_000325).

#### Retrograde labeling of ascending projection neurons in the brainstem

One week after IONC, mice were anesthetized by 1–3% isoflurane, and placed on a stereotaxic apparatus. A small volume (0.15–0.30 µl) of 0.5% cholera toxin B subunit (CTB; C9903; Sigma) in 50 mM PBS was injected into the right V2 VPM (1.60–1.70 mm posterior and 1.80–1.90 mm lateral to the bregma, 3.00–3.20 mm below the dura; Franklin and Paxinos, 2008) through a microneedle syringe (NF35BV and NANOFIL; WPI) by continuous pressure from a microsyringe pump (53311; Stoelting). After ejection, the needle tip was held in the V2 VPM for at least five minutes and then gently retracted to minimize leakage along the track. After 2–4 d of survival, mice were transcardially perfused and transverse brainstem sections (20-µm thickness) were prepared using a freezing microtome. Alternating serial sections were subjected to CTB immunostaining and the remaining were subjected to Nissl and/or CO staining. For fluorescent localization, the sections were incubated successively with 10% normal rabbit serum (NRS) in PBS-X for 30 min, 1:10 000 goat anti-CTB antibody (List Biological catalog #703, RRID:AB_10013220) in PBS-X containing 0.12% λ-carrageenan, 1% NRS, and 0.02% sodium azide (PBS-XCR) overnight, 3.75 µg/ml biotinylated rabbit anti-goat IgG in PBS-XCR (BA-5000; Vector Laboratories) for 2 h, and 2.5 µg/ml Alexa Fluor 633-labeled streptavidin in PBS-X (S21375; Thermo Fisher Scientific) for 2 h. Sections were then mounted, counterstained, and coverslipped.

#### Confocal microscopy

Images of fluorescently stained sections were captured using a Zeiss LSM 710 scanning confocal microscope. Low-magnification images of 7-µm-optical thickness were taken using a 10×/0.45 objective lens (Plan-Apochromat; Carl Zeiss), 0.7× digital zoom, 1.58 µs pixel time, and four times frame average at 1024 × 1024 resolution. High-magnification images of 1-µm-optical thickness were taken using a 63×/1.4 oil immersion lens (Plan-Apochromat; Carl Zeiss), 3.15 µs pixel time, and eight times frame average at 512 × 512 resolution. For quantitative measurements of VGluT2 or VGluT2-MAP2-double immunohistochemistry, two nonoverlapping high-magnification regions of interest (ROIs) were acquired in the left and right V2 VPMs in four alternatively stained sections, respectively. The power of the lasers was 0.4–0.6 mW, which did not induce obvious fading.

#### Image analysis

**Figure 1.**
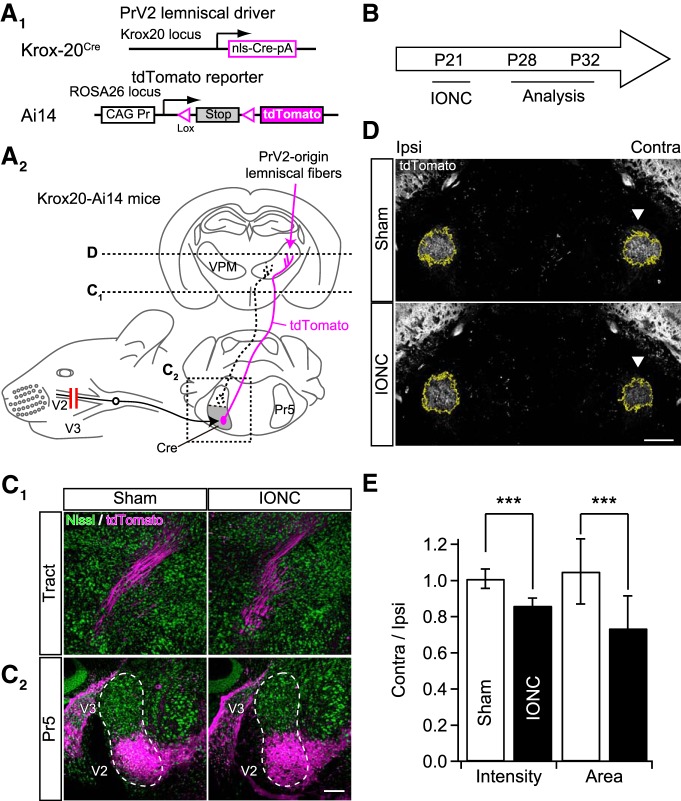
Transection of infraorbital nerve induced anatomic remodeling in whisker sensory pathway. ***A***, Schematic drawing of genetic visualization of PrV2-origin lemniscal fibers. ***A_1_***, Alleles of Krox-20^Cre^ and Ai14 mouse lines. These lines were crossed. ***A_2_***, Selective labeling of PrV2-origin lemniscal fibers with tdTomato. Boxed area over the left Pr5 indicates the region shown in ***C_2_***. Horizontal dotted lines indicate horizontal planes shown in ***C_1_*** and ***D***. ***B***, Experimental schedule of IONC. ***C***, tdTomato signals in the PrV2 (***C_2_***) and on the ascending tract (***C_1_***) did not change after IONC. Scale bar, 200 µm. ***D***, tdTomato signals in the V2 VPM of Krox20-Ai14 mice. Arrowheads indicate the affected V2 VPM. Yellow enclosures indicate automatically detected ROIs for analyses. Scale bar, 500 µm. ***E***, Ratio of contralateral to ipsilateral tdTomato signals of the V2 VPM in fluorescent intensity and area. Values are represented as mean ± SD, 18 images from nine mice were analyzed for each group. Statistical significance was tested by two-way repeated measures ANOVA (main effect of IONC). ***p < 0.001; two-tailed. Pr5, principle trigeminal nucleus; PrV2, maxillary Pr5; V2, maxillary region; V3, mandibular region; VPM, ventral posteromedial thalamic nucleus.

Raw image files were opened using a Carl Zeiss software (ZEN Digital Imaging for Light Microscopy, RRID:SCR_013672), and each image was exported as an 8-bit tiff file. Area and intensity of tdTomato fluorescence in the left and right V2 VPM were measured using Analyze Particles command following auto-binarization by 8-bit, Enhance Contrast, and Threshold commands run as a single macro in ImageJ software (RRID:SCR_003070; Fig. 1*D*). Density and size of VGluT2 puncta were also analyzed using a similar macro except Enhance Contract. The tdTomato-positive and tdTomato-negative VGluT2 puncta in an image were automatically sorted using a macro in Photoshop software (Adobe Photoshop, RRID:SCR_014199), and then subjected to ImageJ analyses. Dendritic and somatic VGluT2 puncta on each MAP2-stained V2 VPM neuron and retrogradely labeled brainstem neurons were manually counted using the Cell Counter plugin of ImageJ. For analysis of dendritic and somatic VGluT2 puncta on MAP2-stained V2 VPM neurons, five well-visualized neurons with large nucleus and thick proximal dendrites were randomly selected in each ROI. For analysis of CTB-labeled neurons, a 0.126 mm^2^ ROI was placed at the center of corresponding brainstem region (e.g., PrV2) for each section and whole CTB-labeled neurons in the ROI were then counted. Four alternatively stained sections for each brainstem region were analyzed for each animal. Images displayed with off-line magnification were processed by upscaling and noise-reduction algorithms (Tomasi and Manduchi, 1998; Freedman and Fattal, 2011) using a house-made Windows software with GeForce technologies (GT 640; NVIDIA; Takeuchi et al., 2014).

#### Identification of maxillary and mandibular regions in the Pr5 and SpI

Maxillary and mandibular regions of the principle trigeminal nucleus, Pr5 (PrV2 and PrV3) were identified by region-specific Cre driver mouse lines, *Krox-20^cre^* and *Hoxa2-cre* (MGI ID: 3773535), respectively (Voiculescu et al., 2000; Ren et al., 2002). These drivers were crossed with tdTomato reporter line (Madisen et al., 2010) or LacZ reporter line (MGI ID: 5587997; Kobayashi et al., 2013). Recombination patterns were the same as previous reports using the same drives to identify PrV2 and PrV3: ventral and dorsal Pr5, corresponding to rhombomere 3 and 2, respectively (Oury et al., 2006; Bechara et al., 2015). Transgenically identified PrV2 and PrV3 regions were well consistent with the regions, where CTB-labeled primary afferent fibers from the whisker pad and lower jaw terminated, respectively. Maxillary and mandibular regions of the interpolar subnucleus of the spinal trigeminal nuclei (V2 SpI and V3 SpI) were also identified by the CTB-labeled primary afferent fiber terminals.

#### Labeling of primary afferent fibers from peripheral tissue to the brainstem

Mice were anesthetized by a ketamine/xylazine cocktail (80/10 mg/kg, i.p.) and placed under a dissecting microscope (type 308795; Wild Heerbrugg). A total of 5 μL of 1% CTB with 0.01% fast green FCF (Chroma-Gesellschaft) in 50 mM PBS was subcutaneously injected into the left whisker pad or the lower jaw through a microneedle syringe (NANOFIL; WPI). After 2–4 d of survival, mice were transcardially perfused, cryo-blocked, sectioned, and stained as described above.

### Electrophysiology

#### Patch-clamp recordings

*Preparation of acute thalamic slices.* Mice were anesthetized with isoflurane (Abbott Japan) and decapitated. Parasagittal 300-µm-thick thalamic slices (Arsenault and Zhang, 2006) were prepared using a vibrating blade microtome (VT1200S; Leica Microsystems) in an ice-cold cutting solution (234 mM sucrose, 2.5 mM KCl, 1.25 mM NaH_2_PO_4_, 10 mM MgCl_2_, 0.5 mM CaCl_2_, 25 mM NaHCO_3_, 0.5 mM *myo*-inositol, and 11 mM glucose) equilibrated with 95%O_2_-5%CO_2_. Slices were recovered in artificial CSF (ACSF; 125 mM NaCl, 2.5 mM KCl, 1.25 mM NaH_2_PO_4_, 1 mM MgSO_4_, 2 mM CaCl_2_, 26 mM NaHCO_3_, and 20 mM glucose) equilibrated with 95%O_2_-5%CO_2_ at 32°C for 30 min and then kept at room temperature. A slice in a recording chamber was perfused by 30–32°C ACSF at a rate of 2.5–3.0 ml min^−1^. During recordings, 10 µM (-)-bicuculline methochloride, 1 µM CGP55845, and 1 µM strychnine were included in the superfusate.

*Whole-cell voltage-clamp recordings of lemniscal EPSCs.* The tip resistance of the patch pipette was 2–5 MΩ when filled with an intracellular solution: 120 mM CsMeSO_3_, 10 mM HEPES, 1 mM EGTA, 2 mM MgCl_2_, 0.1 mM CaCl_2_, 20 mM NaCl, 5 mM QX-314, 2 mM ATP-Na_2_, 0.5 mM GTP-Na, and 0.5% biocytin, pH 7.3, 290–300 mOsm. Liquid junction potential was not compensated. Recordings were made from V2 VPM neurons under the infrared-differential interference contrast view of an upright microscope as previously described (Takeuchi et al., 2014). Recordings were performed using a MultiClamp700A amplifier (MDS) and an ITC-18 AD/DA board (HEKA Elektronik) with IGOR Pro. Signals were filtered at 3 kHz and digitized at 50 kHz. The series resistance was compensated. If series resistance varied by > 20% or increased above 20 MΩ, the data were discarded. Data analysis was performed using IGOR Pro.

*Recordings of lemniscal fiber-mediated synaptic currents*. Lemniscal fiber-mediated synaptic currents were recorded as previously described (Takeuchi et al., 2012). Briefly, a concentric bipolar electrode was placed on the medial lemniscal fiber bundle and electrical square pluses were then delivered at 0.1 Hz (100-μs duration, typically 10–400 μA). Paired-pulse depression of EPSCs were recorded. Evaluation of the number of lemniscal fibers innervating a recorded V2 VPM neuron was estimated by counting stepwise increments of EPSC amplitude in response to increasing stimulus intensity. Lemniscal fiber-mediated miniature EPSCs were recorded in modified ACSF, substituting Sr^2+^ ions for Ca^2+^ ions in the presence of 100 µM _DL_-APV at -90 mV. Test stimulations were delivered at 0.1 Hz. The miniature EPSCs were detected and analyzed by a semi-automated IGOR Pro procedure (Takeuchi et al., 2012).

#### In vivo extracellular recordings of V2 VPM neurons

One to two weeks or one to three months after IONC, mice were anesthetized with 1–3% isoflurane, and placed on a stereotaxic apparatus. Rectal temperature was maintained at 36–37°C using a DC temperature controller (40-90-8D; FHC). Stages of anesthesia were maintained by confirming the lack of vibrissae movements and eyelid reflex (Friedberg et al., 1999; Miyata et al., 2003). The head position was adjusted until the heights of the bregma and the lambda became equal. A small craniotomy was operated at the skull above the right V2 VPM, and a 16-channel silicon probe (A1x16-5mm-25-177 or A1x16-Poly2-5mm-50s; NeuroNexus) or a single microelectrode (2–4 MΩ; FHC or Unique Medical) was vertically inserted into the V2 VPM using a stepper motor manipulator (1.60–1.90 mm posterior and 1.70–2.10 mm lateral to the bregma, 3.00–3.50 below the dura; Franklin and Paxinos, 2008). A screw-type reference electrode (TN204-089B, Unique Medical) was fixed in the rostral skull. Receptive fields of V2 VPM neurons were identified as multi-unit neural firings in response to tactile stimuli throughout the body. This receptive field mapping was conducted at 50-µm interval along electrode track. At each recording site, the receptive fields were roughly surveyed at first using paint brushes, metal probes (FST), and air-puff through a 26-G needle controlled by a pneumatic pico pump (PV830; WPI). The size and precise location of each receptive field was then identified using von Frey filaments (Semmes-Weinstein monofilaments; Stoelting), and/or a piezoelectric device (M-2629B; MESS-TEK; Simons, 1983) if it included large whiskers. The timing of these stimuli was monitored using a CMOS camera (MQ003CG-CM; Ximea). Neural activity was processed with an OmniplexD 16 ch system (Plexon) or an extracellular amplifier (2400A; Dagan), filtered at 0.25–10 kHz, and digitized at 40–50 kHz with OmniPlex software (RRID:SCR_014803) or Spike2 software (RRID:SCR_000903). After recordings, several recording sites were marked by feeding anodal currents through the electrode (typically 5 µA, 10 s). The mice were then deeply anesthetized with pentobarbital (120 mg/kg, i.p.) and transcardially perfused with ice-cold saline followed by 4% PFA and 0.2% picric acid in 0.1 M PB. The brains were postfixed overnight, then coronally sectioned at 50-µm thickness using a vibrating blade microtome (VT1000S; Leica Microsystems). The sections were then mounted, counterstained with fluoro-Nissl staining, and coverslipped. Images were taken using a confocal laser scanning microscope as described above. Recording sites were reconstructed using Neurolucida software. Only recordings made in the V2 VPM, which was easily recognized by strong tdTomato fluorescence from PrV2-origin lemniscal fibers, were analyzed. Spikes were detected and sorted by custom software written in MATLAB (RRID:SCR_001622) or IGOR Pro (Quiroga et al., 2004).

### Tactile sensory test

Mice were habituated to being held in the experimenter’s hands and then to von Frey filament application to their maxillary and mandibular regions one or two week(s) before IONC (Seino et al., 2009). On P21 or P60, the ION was cut or sham operated as described above. To evaluate escape thresholds, quantitative mechanical stimuli via von Frey filaments with approximately equal logarithmic bending forces (0.02-, 0.03-, 0.07-, 0.16-, 0.4-, 0.6-, 1.0-, 1.4-, and 2.0-g forces, respectively) were applied to the maxillary and mandibular regions in ascending order. Each von Frey filament was applied five times. If mice showed positive withdrawal responses, e.g., brisk withdrawal of the head, face-grooming strokes directed to the stimulated facial area, at least three times, the bending force of that filament was defined as the escape threshold. If mice did not show any responses to 2.0 g of force, the escape threshold was set at 2.0 g (cutoff). This series of assessment was repeated three times and the average of the three withdrawal thresholds was recorded for that day.

### Statistical analysis

Unless otherwise noted, all values are given as means ± SD between observations. Means ± SD between animals were also given in the text as parenthesized. Appropriate statistical tests were performed depending on experimental designs; for example when eight nonoverlapping image acquisitions were made on brain sections from each sham or IONC mouse, two-way repeated measures ANOVA was employed to extract major effect of operation or survival time on measured values (the other effect was animal; Glantz, 2011). Details of statistical analyses were given in respective results, figure legends, and Table 1 (statistical table). *Post hoc* power analysis was performed using G*Power3 software (http://www.gpower.hhu.de/; RRID:SCR_013726; Faul et al., 2007). R software was applied for statistical tests (R Project for Statistical Computing, RRID:SCR_001905). The significance level was set at *p* < 0.05.

**Table 1.** Statistical tests

	Data structure	Type of test	Power (α = 0.05)
a (Fig. 1E, intensity)	Normal	Two-way repeated measures ANOVA	1.0000000
a (Fig. 1E, area)	Normal	Two-way repeated measures ANOVA	1.0000000
b [Fig. 2B, tdT(+)]	Normal	Two-way repeated measures ANOVA	1.0000000
c [Fig. 2B, tdT(-)]	Unknown	Two-way repeated measures ANOVA	1.0000000
d	Normal	Two-way repeated measures ANOVA	0.9786688
e (Fig. 2C)	Normal	Two-way repeated measures ANOVA	1.0000000
f [Fig. 2D, soma, tdT(+)]	Unknown	Two-way repeated measures ANOVA	1.0000000
f [Fig. 2D, dendrites, tdT(+)]	Unknown	Two-way repeated measures ANOVA	1.0000000
g [Fig. 2D, soma, tdT(-)]	Unknown	Two-way repeated measures ANOVA	1.0000000
g [Fig. 2D, dendrites, tdT(-)]	Unknown	Two-way repeated measures ANOVA	1.0000000
h	Unknown	Pearson’s χ^2^ test	0.8460473
i (Fig. 2F)	Unknown	*t* test for noncorrelation	0.9759325
j [Fig. 2I, tdT(+)]	Normal	Two-way repeated measures ANOVA	0.2974602
j [Fig. 2I, tdT(-)]	Normal	Two-way repeated measures ANOVA	0.1588404
j [%tdT(+)]	Unknown	Two-way repeated measures ANOVA	0.2390769
k	Unknown	Two-way repeated measures ANOVA	1.0000000
l (Fig. 3B)	Unknown	Kolmogorov-Smirnov two-sample test	n.a. (*p* = 5.5 × 10^−28^)
m	Normal	Two-way repeated measures ANOVA	0.1673505
n (Fig. 3D)	Unknown	Kolmogorov-Smirnov two-sample test	n.a. (*p* = 9.0 × 10^−83^)
o (Fig. 4C2, Bouton)	Normal	Two-way repeated measures ANOVA	1.0000000
o (Fig. 4C2, Terminal)	Normal	Two-way repeated measures ANOVA	1.0000000
o (Fig. 4C2, Branch)	Normal	Two-way repeated measures ANOVA	1.0000000
p (Fig. 4D2 right)	Unknown	Kolmogorov-Smirnov two-sample test	n.a. (*p* = 1.7 × 10^−7^)
q (Fig. 4D2 left)	Unknown	Two-way repeated measures ANOVA	0.9999438
r (Fig. 4E2 left)	Unknown	Kolmogorov-Smirnov two-sample test	n.a. (*p* = 2.7 × 10^−148^)
r (Fig. 4E2 right)	Unknown	Kolmogorov-Smirnov two-sample test	n.a. (*p* = 2.7 × 10^−115^)
s (Fig. 5D, PrV2)	Normal	Two-way repeated measures ANOVA	1.0000000
s (Fig. 5D, PrV3)	Unknown	Two-way repeated measures ANOVA	1.0000000
s (Fig. 5D, V2 SpI)	Normal	Two-way repeated measures ANOVA	0.9465853
s (Fig. 5D, V3 SpI)	Normal	Two-way repeated measures ANOVA	1.0000000
s (Fig. 5D, DCN)	Normal	Two-way repeated measures ANOVA	1.0000000
t	Unknown	Two-way repeated measures ANOVA	1.0000000
u (Fig. 6E right)	Normal	Two-way repeated measures ANOVA	0.8840900
v (Fig. 7D, 1 m intensity)	Normal	Two-way repeated measures ANOVA	1.0000000
v (Fig. 7D, 1 m area)	Normal	Two-way repeated measures ANOVA	1.0000000
v (Fig. 7D, 3 m intensity)	Normal	Two-way repeated measures ANOVA	1.0000000
v (Fig. 7D, 3 m area)	Normal	Two-way repeated measures ANOVA	1.0000000
w	Normal	Two-way repeated measures ANOVA	1.0000000
x	Normal	Two-way repeated measures ANOVA	0.5385280
y [Fig. 7E, 3 m, tdT(+)]	Normal	Two-way repeated measures ANOVA	1.0000000
z [Fig. 7E, 3 m, tdT(-)]	Normal	Two-way repeated measures ANOVA	1.0000000
aa [Fig. 7F, 3 m, %tdT(+)]	Normal	Two-way repeated measures ANOVA	1.0000000
bb (Fig. 7I bottom)	Normal	Two-way repeated measures ANOVA	1.0000000
cc (Fig. 8C, intensity)	Normal	Two-way repeated measures ANOVA	1.0000000
cc (Fig. 8C, area)	Normal	Two-way repeated measures ANOVA	1.0000000
dd [Fig. 8E, tdT(+)]	Normal	Two-way repeated measures ANOVA	1.0000000
ee [Fig. 8E, tdT(-)]	Normal	Two-way repeated measures ANOVA	1.0000000
ff	Normal	Two-way repeated measures ANOVA	0.1898718
gg [Fig. 8F, contra]	Normal	Two-way repeated measures ANOVA	1.0000000

Each small alphabetical character indicates statistical tests labeled with a *p* value and the small alphabetical character in the Results section. Data normality was determined using the Lilliefors test. Nonparametric statistical analysis produced similar results. *Post hoc* power analysis was performed using G*Power 3 software (Faul et al., 2007). *Post hoc* powers of two-way repeated measures ANOVA were calculated on main effect of operation (IONC or whisker deprivation) except w and x (main effect of survival period). n.a., not applicable; *p* values were provided instead.

## Results

### Injury of primary whisker sensory nerve decreased PrV2-origin lemniscal fibers in V2 VPM

To selectively assess anatomic remodeling of the PrV2-origin lemniscal fibers, we employed a Krox20-Ai14 mouse line (Fig. 1*A*), in which most of the projection neurons in the PrV2 specifically express tdTomato (Takeuchi et al., 2014). In Krox20-Ai14 mice, tdTomato was specifically found in the V2 region of the Pr5 (PrV2), but not in the V3 region (PrV3; Fig. 1*C_2_*) or other trigeminal nuclei (data not shown). IONC (Fig. 1*B*) did not alter this specific expression pattern (Fig. 1*C_2_* ). Projection neurons in the PrV2 were thoroughly visualized from the soma to synaptic terminals in the V2 VPM (Fig. 1*C*,*D*). Unilateral IONC significantly decreased macroscopically observed tdTomato signal intensity and the occupying area in the contralateral V2 VPM [contra/ipsi ratio: 0.86 ± 0.04 and 0.74 ± 0.18 (0.86 ± 0.06 and 0.77 ± 0.26 between animals) for intensity and area, respectively, ***p < 0.001^a^, main effect of IONC, *F*_(1,18)_ > 52.3 for both; Fig. 1*D*,*E*]. These results suggest that IONC significantly reduces the PrV2-origin lemniscal fiber territory in the V2 VPM.

### IONC increased non-PrV2-origin lemniscal fiber terminals in V2 VPM related to multiple lemniscal fiber innervations onto a V2 VPM neuron

**Figure 2.**
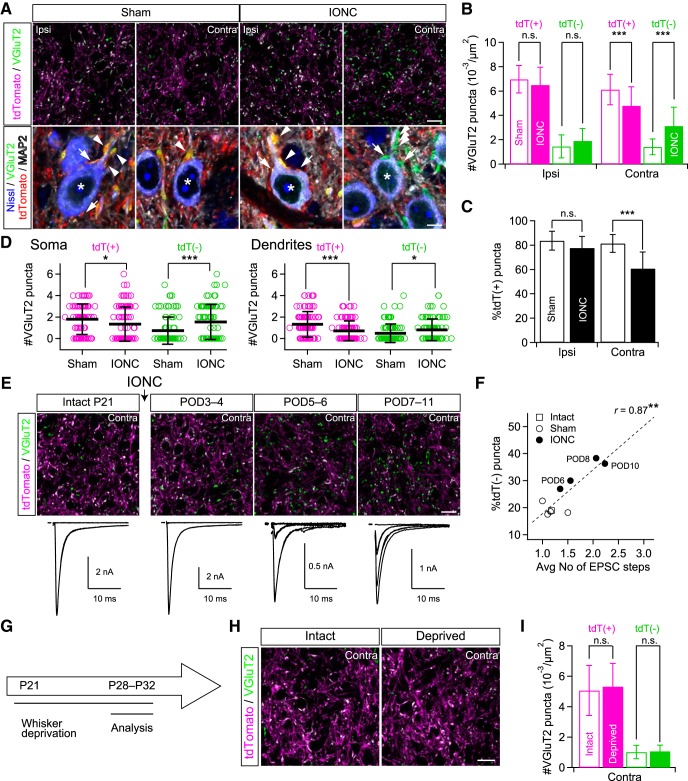
IONC increased non-PrV2-origin lemniscal fiber terminals in V2 VPM related to newly recruited lemniscal fibers onto VPM neurons. ***A***, VGluT2-immunostained V2 VPM sections of Krox20-Ai14 mice. VGluT2 is a marker of lemniscal fiber terminals in the VPM. Top, Significant increase of tdT-negative (non-PrV2-origin) VGluT2 puncta in the contralateral V2 VPM after IONC. Scale bar, 20 µm. Bottom, The soma and dendrites of V2 VPM neurons were visualized by fluoro Nissl and MAP2 stainings, respectively, in addition to VGluT2 and tdTomato (top) to examine subcellular distribution of the VGluT2 puncta in their origin-specific manner. * indicates a V2 VPM neuron; arrows indicate somatic puncta; arrowheads indicate dendritic puncta. Scale bar, 5 µm. ***B***, Summary density of VGluT2 puncta in the V2 VPM. tdT(+) = tdTomato-positive VGluT2; tdT(-) = tdTomato-negative ones. ***C***, IONC decreased proportion of tdT(+) VGluT2 puncta in the contralateral V2 VPM. ***D***, Summary numbers of VGluT2 puncta on the soma (left) and proximal dendrites (right) in the contralateral V2 VPM. tdT-positive puncta decreased whereas tdT-negative ones increased both on the soma and dendrites. A marker indicates each V2 VPM neuron examined. tdT(+) = tdTomato-positive VGluT2; tdT(-) = tdTomato-negative ones. ***E***, Correlated postoperative time course of anatomic and functional lemniscal fiber remodeling in the V2 VPM. Top: VGluT2 immunostaining. Scale bar, 20 µm. Bottom: Discrete lemniscal fiber-mediated EPSCs in a V2 VPM neuron in response to increasing electrical stimuli on the medial lemniscal fiber bundle (Takeuchi et al., 2012). ***F***, Correlation between anatomic and functional lemniscal fiber remodeling in the contralateral V2 VPM. *r*, Pearson’s correlation coefficient. Broken line is the regression line. ***G***, Experimental schedule of whisker deprivation. ***H***, VGluT2-immunostained sections of the V2 VPM in Krox20-Ai14 mice after whisker deprivation. Scale bar, 20µm. ***I***, Summary density of VGluT2 puncta in the contralateral V2 VPM. tdT(+) = tdTomato-positive VGluT2; tdT(-) = tdTomato-negative ones. Values are represented as mean (±SD). Three to nine mice (eight images from each mouse) were analyzed for each group in ***A–I***. In total, 94 neurons from four mice (4-10 neurons from each image) were analyzed for each group in ***D***. In total, 215 neurons from 91 mice and 118 images from 14 mice were analyzed for ***E*** and ***F***. Statistical significance was tested by two-way repeated measures ANOVA (main effect of IONC or whisker deprivation; **B–D** and ***I***) and Pearson’s product-moment correlation test (***F***). *p < 0.05; **p < 0.01; ***p < 0.001; n.s., not significant; two-tailed.

We have previously demonstrated that complete transection of ION newly recruits additional multiple lemniscal fibers onto a V2 VPM neuron (multiple innervation; Takeuchi et al., 2012). To reveal somatotopic reorganization underling this rewiring, we conducted VGluT2-immunohistochemistry in the V2 VPM of Krox20-Ai14 mice after IONC; VGluT2 is a marker of lemniscal fiber terminals (Graziano et al., 2008). Previously, we have shown that in the V2 VPM of Krox20-Ai14 mice, PrV2-origin and non-PrV2-origin lemniscal fiber terminals are represented as tdTomato (tdT)-positive and -negative VGluT2 puncta, respectively (Takeuchi et al., 2014). Here, we found that, after IONC, the density of tdT-positive VGluT2 puncta in the contralateral V2 VPM significantly decreased [111.4 ± 9.7 and 87.3 ± 14.1 (111.4 ± 27.5 and 87.3 ± 39.8 between animals) in 18,212 µm^2^ ROI for sham and IONC, respectively; ***p < 0.001^b^, main effect of IONC, *F*_(1,222)_ = 33.1], whereas the density of tdT-negative ones increased [25.7 ± 11.8 and 57.1 ± 28.1 (25.6 ± 14.8 and 57.1 ± 44.0) in 18,212 µm^2^ ROI; ***p < 0.001^c^, *F*_(1,222)_ = 73.0; Fig. 2*A*,*B*]. The total density of VGluT2 puncta in the contralateral V2 VPM (tdT-positive + tdT-negative) did not differ between sham and IONC groups [137.1 ± 26.2 and 144.3 ± 40.7 (137.1 ± 38.1 and 144.3 ± 76.4) in 18,212 µm^2^ ROI; *p* = 0.221^d^, *F*_(1,222)_ = 1.5]. Thus, the percentage of tdT-positive VGluT2 puncta in the contralateral V2 VPM significantly decreased after IONC [81.3 ± 7.4% and 60.8 ± 13.5% (81.3 ± 6.7 and 60.8 ± 14.7); ***p < 0.001^e^, *F*_(1,222)_ = 102.2; Fig. 2*C*]. These results indicate considerable retraction of PrV2-origin lemniscal fibers and invasion of non-PrV2-origin ones in the V2 VPM after IONC.

Given the results above, we then examined whether IONC increases the number of contacts between non-PrV2-origin VGluT2 puncta and a V2 VPM neuron. VGluT2 puncta could be observed both on the soma and proximal dendrites of V2 VPM neurons as previously reported (Matthews and Faciane, 1977; Liu et al., 1995; Takeuchi et al., 2014; Fig. 2*A*, bottom). In the contralateral V2 VPM of the IONC group, tdT-negative puncta formed contacts on V2 VPM neurons, and their numbers on each V2 VPM neuron in 1-µm-thick optical section significantly increased on both the soma and dendrites [soma: 0.73 ± 1.26 and 1.54 ± 1.63 (0.69 ± 1.30 and 1.54 ± 2.75 between animals) for sham and IONC, respectively; dendrites: 0.49 ± 0.86 and 0.81 ± 1.01 (0.49 ± 0.71 and 0.82 ± 2.04); *p < 0.018^f^, main effect of IONC, *F*_(1,180)_ > 5.7 for both; Fig. 2*A*, bottom, Fig. 2*D*]. In contrast, both somatic and dendritic tdT-positive VGluT2 puncta significantly decreased [soma: 1.80 ± 1.42 and 1.34 ± 1.60 (1.78 ± 1.10 and 1.35 ± 2.74); dendrites: 1.32 ± 1.19 and 0.71 ± 0.92 (1.37 ± 1.48 and 0.71 ± 1.60); *p < 0.037^g^, *F*_(1,180)_ > 4.4; Fig. 2*A*, bottom, Fig. 2*D*]. These results suggest that after IONC, newly recruited non-PrV2-origin fibers form new synaptic contacts both on the soma and dendrites of V2 VPM neurons while PrV2-origin fibers retract from preexisting contacts. When we classified each V2 VPM neuron by the origin of converging afferent fibers on the neuron, in the control conditions, 76–77% of V2 VPM neurons received only tdT-positive VGluT2 puncta on the soma or proximal dendrites, 22–23% of V2 VPM neurons received only tdT-negative VGluT2 puncta, but only 1% of V2 VPM neurons received both tdT-positive and -negative ones. IONC decreased the proportion of V2 VPM neurons receiving only tdT-positive VGluT2 puncta to 46% and increased the proportion of those that received only tdT-negative ones to 52% [*p* = 0.004^h^, χ^2^ = 10.81 (*n* = 188 neurons)]. Even after IONC, V2 VPM neurons receiving both tdT-positive and -negative ones were still rare (2%).

Next, we examined the correlation between the emergence of tdT-negative VGluT2 puncta and electrophysiologically assessed multiple innervation of lemniscal fibers onto a V2 VPM neuron after IONC (Takeuchi et al., 2012). On postoperative day (POD)5–POD6, the number of lemniscal fiber-mediated EPSC steps in a V2 VPM neuron increased and became statistically significant (Fig. 2*E*, bottom; Takeuchi et al., 2012). tdT-negative VGluT2 puncta also increased with a time course matching the EPSC steps (Fig. 2*E*). Importantly, the percentage of tdT-negative VGluT2 puncta and EPSC steps were significantly correlated (*r* = 0.87, **p = 0.002^i^, *t*_7_ = 4.63; Fig. 2*F*). Here, we also explored mechanisms underlying lemniscal fiber remodeling in the V2 VPM. Whisker deprivation from P21 did not decrease tdT-positive VGluT2 puncta [92.2 ± 29.9 and 97.0 ± 27.7 (105.8 ± 69.8 and 97.0 ± 12.9 between animals) in 18,212 µm^2^ ROI for intact and whisker deprivation, respectively], increase tdT-negative ones [18.6 ± 8.0 and 19.5 ± 7.1 (18.2 ± 11.4 and 19.5 ± 7.4) in 18,212 µm^2^ ROI], or change the percentage of tdT-positive ones [81.3 ± 12.9% and 82.7 ± 6.7% (83.4 ± 21.0% and 82.7 ± 8.2%)] in the contralateral V2 VPM (*p* > 0.549^j^, main effect of whisker deprivation, *F*_(1,36)_ < 0.4 for all; Fig. 2*G*–*I*). Importantly, whisker deprivation does not recruit multiple lemniscal fiber innervations (Takeuchi et al., 2012), suggesting an injury-induced, but not sensory experience-dependent, mechanism. Hence these results suggest that multiple lemniscal fiber innervations onto a V2 VPM neuron after IONC are mediated at least partially by newly recruited lemniscal fibers of non-PrV2-origin and the extent of the multiple innervation is predictable by anatomic assessments using Krox20-Ai14 mice.

### IONC decreased size of PrV2-origin lemniscal fiber terminals in V2 VPM and amplitude of lemniscal fiber-derived miniature EPSCs

**Figure 3.**
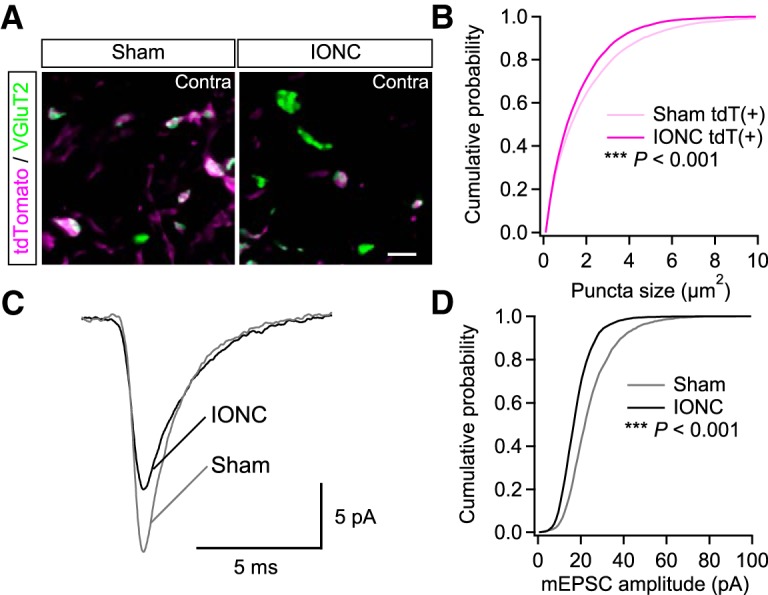
IONC decreased size of PrV2-origin lemniscal fiber terminals in V2 VPM and amplitude of lemniscal fiber-derived miniature EPSCs. ***A***, VGluT2-immunostained V2 VPM sections of Krox20-Ai14 mice. Scale bar, 5 µm. ***B***, Cumulative probabilities of tdT-positive VGluT2 puncta in the V2 VPM after sham and IONC operations. Nine mice (eight images from each mouse) were analyzed for each group. ***C***, Averaged traces of asynchronously released miniature EPSCs from a lemniscal fiber in the VPM. Traces of 1517 and 2356 events from 12 and 15 neurons of sham- and IONC-operated mice, respectively. ***D***, Cumulative probabilities of lemniscal fiber-derived miniature EPSC amplitude. Statistical significance was tested by two-sample Kolmogorov-Smirnov test (***B*** and ***D***). ***p < 0.001; two-tailed.

To estimate the strength of lemniscal fiber synapses specifically after IONC, we measured the sizes of tdT-positive and -negative VGluT2 puncta in the contralateral V2 VPM (Fig. 3). IONC operation selectively decreased the size of tdT-positive VGluT2 puncta [2.28 ± 0.20 and 2.05 ± 0.24 µm^2^ (2.28 ± 0.123 and 2.02 ± 0.29 µm^2^ between animals) for sham and IONC; *p* < 0.001^k^, main effect of IONC, *F*_(1,222)_ = 21.2] and the distributions of sham and IONC groups were significantly different (***p < 0.001^l^, *D* = 0.08; Fig. 3*A*,*B*). However, sizes of tdT-negative ones were not different between sham and IONC groups [2.05 ± 0.31 vs 2.05 ± 0.24 µm^2^ (2.05 ± 0.37 and 2.05 ± 0.18 µm^2^); *p* = 0.697^m^, *F*_(1,222)_ = 0.152]. Consistent with these anatomic results, the amplitude of miniature EPSCs evoked by electrical stimulation of the lemniscal fiber bundle significantly decreased after IONC (***p < 0.001^n^, *D* = 0.28; Fig. 3*C*,*D*; Takeuchi et al., 2012). These results suggest fiber-origin-specific weakening of lemniscal fiber synapses after IONC.

### IONC induced morphologic changes of PrV2-origin lemniscal fibers in V2 VPM

**Figure 4.**
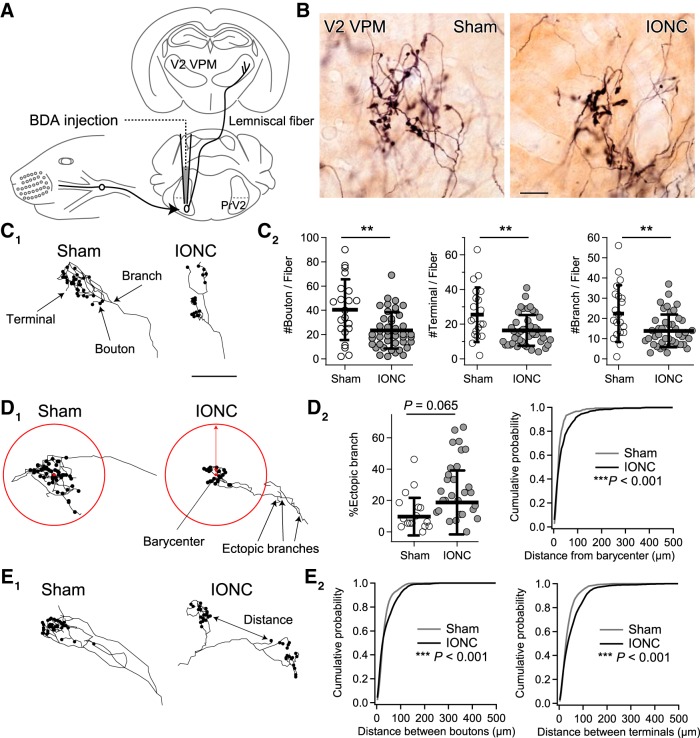
IONC induced morphologic changes of PrV2-origin lemniscal fibers in V2 VPM. ***A***, BDA, an anterograde neural tracer, was injected in the left PrV2. ***B***, PrV2-origin lemniscal fibers in the contralateral V2 VPM. Scale bar, 10 µm. ***C–E***, Projection images of 3-D reconstructed terminal morphology of single PrV2-origin lemniscal fibers (***C_1_***, ***D_1_***, and ***E_1_***) and their quantitative analyses (***C_2_***, ***D_2_***, and ***E_2_***). ***C***, Scale bar, 50 µm. ***D***, Ectopic branches were defined as branches over 50 µm away from the barycenter of boutons. ***E***, Distances between all pairs of boutons and terminals in each PrV2-origin fiber were analyzed. Values are represented as mean ± SD. A marker indicates each fiber (***C_2_***, and ***D_2_*** left). Twenty-one and 44 fibers from three mice were analyzed for sham and IONC groups, respectively. Statistical significance was tested by two-way repeated measures ANOVA (main effect of IONC; ***C_2_***, and ***D_2_*** left) and two-sample Kolmogorov-Smirnov test (***D_2_*** right, and ***E_2_***). **p < 0.01; ***p < 0.001; two-tailed.

To establish a precise anatomic basis for lemniscal fiber remodeling after IONC, we visualized individual PrV2-origin lemniscal fibers at single axon resolution using an anterograde tracer BDA (Fig. 4*A*,*B*). Each single PrV2-origin lemniscal fiber was serially traced, reconstructed, and quantitatively analyzed (Fig. 4*C–E*). In the sham group, each PrV2-origin lemniscal fiber proceeded from caudal to rostral in the V2 VPM with few branches, and then exhibited bushy terminal arborization with clustered numerous axon terminals and synaptic boutons within 100 µm in diameter (Fig. 4*B*,*C_1_*–*E_1_*). After IONC, the numbers of boutons [23.4 ± 15.0 between fibers (18.6 ± 17.3 between animals)], terminals [16.4 ± 8.9 (14.5 ± 4.4)], and branches [13.8 ± 8.1 (12.7 ± 3.1)] within each fiber were all significantly decreased compared with those of the sham group [40.5 ± 25.0 (50.9 ± 17.4), 25.4 ± 15.7 (32.7 ± 12.3), and 22.3 ± 14.1 (28.1 ± 9.7), respectively; **p < 0.005°, main effect of IONC, *F*_(1,59)_ > 8.5 for all; Fig. 4*C_2_*]. IONC also made axon branches much farther away from the barycenter of terminal arborization (***p < 0.001^p^, *D* = 0.17; Fig. 4*D*_2_, right). If branches >50 µm away from the barycenter are defined as ectopic branches, then the percentage of ectopic branches within each PrV2-origin lemniscal fiber tended to increase after IONC [18.8 ± 20.4% (25.9 ± 22.7%)] compared with that in the sham group [9.6 ± 12.0% (9.5 ± 6.2%); *p* = 0.065^q^, *F*_(1,59)_ = 3.5; Fig. 4*D*_2_, left]. Furthermore, IONC increased the distances between bouton pairs and terminal pairs within each PrV2-origin lemniscal fiber, indicating diverging innervation patterns (***p < 0.001^r^, *D* > 0.13 for both; Fig. 4*E*). These results indicate that each PrV2-origin lemniscal fiber is weakened but has diverging innervation patterns in the V2 VPM after IONC.

### IONC induced additional lemniscal fiber innervations from non-V2 brain stem nuclei to V2 VPM

**Figure 5.**
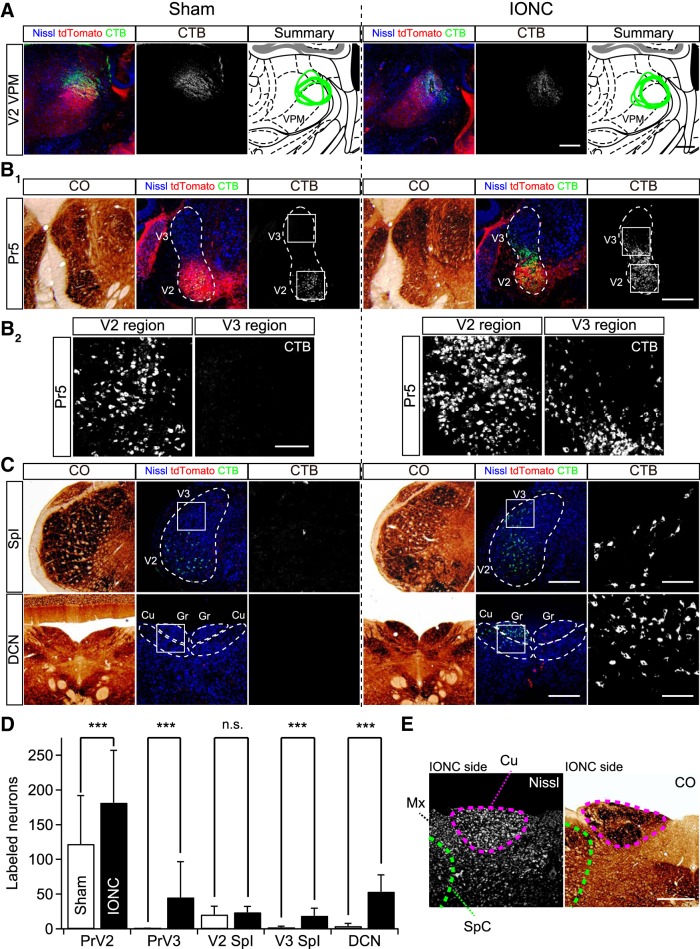
IONC induced lemniscal fiber innervations from non-V2 brainstem nuclei to V2 VPM. ***A***, A solution containing CTB, an efficient neural tracer, was injected in the right V2 VPM of sham (left)- or IONC (right)-operated Krox20-Ai14 mice to retrogradely label projection neurons terminating the V2 VPM. Injection sites of analyzed mice were reconstructed as green enclosures on the brain atlas of Franklin and Paxinos (2008). Injection sites shown as pictures were depicted by thick lines. Scale bars, 400 µm. ***B***, Retrogradely labeled neurons in the V2 and V3 regions of the Pr5 in sham (left) and IONC (right). See Materials and Methods for details regarding identification of V2 and V3 regions. CO-stained adjacent sections are also shown (***B_1_***, leftmost). Squared regions in ***B_1_*** are magnified in ***B_2_***. Scale bars for low (***B_1_***) and high (***B_2_***) magnifications, 400 and 100 µm, respectively. ***C***, Retrogradely labeled neurons in the interpolar subnucleus of the spinal trigeminal nuclei (SpI), and the DCNs in sham (left) and IONC (right). CTB-immunofluorescence of squared regions is magnified in the rightmost and CO-stained adjacent sections are also shown (leftmost). Scale bars for low and high magnifications, 400 and 100 µm. Cu, cuneate nucleus; Gr, gracile nucleus. ***D***, Summary bar graph showing numbers of retrogradely labeled neurons from the V2 VPM. Values are represented as mean ± SD. Three and four mice (four ROIs of separate sections from each animal) were examined for sham and IONC groups, respectively. Statistical significance was tested by two-way repeated measures ANOVA (main effect of IONC). ***p < 0.001; n.s., not significant; two-tailed. ***E***, Nissl- and CO-stained adjacent brainstem sections of an IONC-operated mouse showing Cu and the caudal subnucleus of the spinal trigeminal nuclei (SpC) in the ipsilateral side of IONC. Clear cyto-architecture was retained; there is no collapse of boundaries between Cu and SpC. Mx, matrix region of the medulla. Scale bar, 400 µm.

What are the origins of the non-PrV2-origin lemniscal fiber terminals in the V2 VPM after IONC? To address this question, CTB, a retrograde neural tracer, was locally injected in the V2 VPM as described previously (Takeuchi et al., 2014; Fig. 5*A*). In the sham group, retrogradely labeled projection neurons in the brainstem were observed almost exclusively in the contralateral PrV2 and V2 SpI [122.2 ± 69.5 and 20.7 ± 11.9 (122.1 ± 161.2 and 20.7 ± 26.8 between animals), respectively; three mice (four 0.126 mm^2^ ROIs from separate sections from each mouse); Fig. 5*B*–*D*]. Surprisingly, after IONC, additionally significantly labeled neurons were observed in the PrV3, V3 SpI, and dorsal column nuclei (DCNs) [45.4 ± 51.4, 18.8 ± 10.8, and 53.6 ± 24.0 (45.4 ± 109.0, 18.8 ± 20.6, and 53.6 ± 52.3), respectively; ***p < 0.001^s^, main effect of IONC, *F*_(1,21)_ > 74.4 for all, four mice], whereas retrograde labelings in those nuclei were negligible in the sham group [0.3 ± 0.7, 2.4 ± 1.4, and 4.2 ± 3.9 (0.3 ± 1.2, 2.4 ± 2.6, and 4.2 ± 5.1), respectively; Fig. 5*B*–*D*]. The retrogradely labeled neurons in such ectopic regions were all tdT-negative. Ipsilateral labelings were not observed in the sham or IONC group. After IONC, labeled neurons increased also in the caudal subnucleus of the spinal trigeminal nuclei (SpC) although the number was still relatively small [1.2 ± 1.4 and 5.5 ± 2.4 (1.2 ± 3.2 and 5.5 ± 3.2) for sham and IONC, respectively; *p* < 0.001^t^, main effect of IONC, *F*_(1,21)_ = 49.2, data not shown]. These results indicate that the origins of non-PrV2-origin lemniscal fiber terminals in the V2 VPM after IONC include V3 subregions of the trigeminal nuclei and DCN. Although ectopic innervations from even nontrigeminal brainstem nuclei such as the DCN to the V2 VPM after IONC were suggested, normal cyto-architecture and delineation were maintained over the trigeminal nuclei and the DCN (Fig. 5*B_1_*, *C*). There was no collapse of boundaries between trigeminal nuclei and DCN after IONC even at the closest site between them (Fig. 5*E*).

### Alterations in receptive fields and behavior after IONC

**Figure 6.**
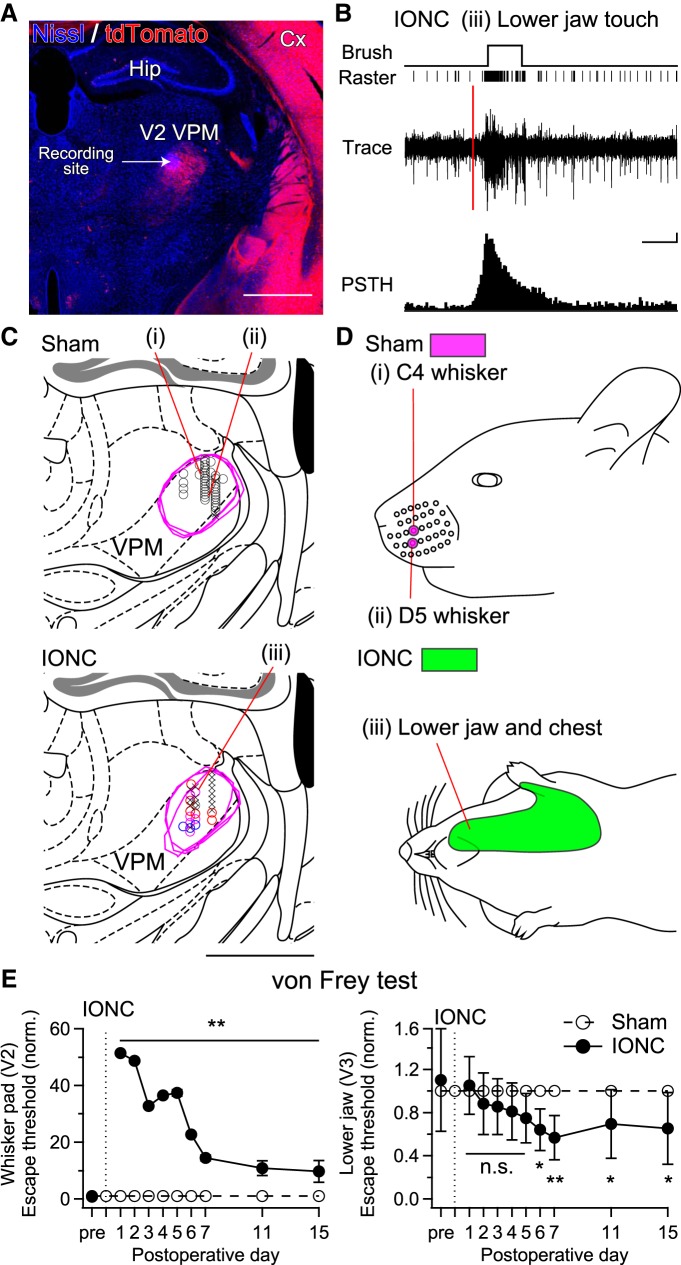
IONC induced ectopic receptive fields of V2 VPM neurons and mechanical hypersensitivities on mandibular region of face. ***A***, **C**, Recording sites located by electrical lesion made after receptive field recordings. Only recordings in the V2 VPM characterized by dense tdTomato-labeled lemniscal fiber terminals were analyzed. A representative image is shown (***A***), and all recording sites analyzed were reconstructed on the brain atlas of Franklin and Paxinos (2008) (***C***). Black circles = recording sites of maxillary receptive fields only; blue circles = recording sites of mandibular receptive fields; red circles = recording sites of nontrigeminal receptive fields; Magenta circles = recording sites of both mandibular and nontrigeminal receptive fields; multiplication signs = nonresponsive sites. Magenta brackets indicate the manually traced V2 VPM regions. Roman numerals indicate recording sites of representative recordings shown in ***B*** and ***D***. Scale bars, 1 mm. Cx, cerebral cortex; Hip, hippocampus; VPM, ventral posteromedial thalamic nucleus. ***B***, Representative recording of IONC-operated mouse at the recording site (iii). Red vertical line indicates the timing of cue for stimulation. Time scale, 250 ms; Scale for peristimulus time histogram, 10 spikes. ***D***, Representative receptive fields of V2 VPM neurons. Each Roman numeral corresponds to the recording from the site indicated in ***C***. Colored areas on the age-matched mouse traces indicate receptive fields at the recording sites. Magenta = sham; green = IONC. Body surface at which there is stimulus-locked significant increase in firing rate from basal firing is considered as the receptive field. ***E***, Face-escape thresholds assessed by von Frey filament stimuli on whisker pad and lower jaw before and 1–15 d after IONC operation on P21. Each value was normalized to that of sham group. Twelve mice for each group were examined. Values are represented as mean ± SD. Statistical significance was tested by two-way repeated ANOVA and *post hoc* Tukey HSD test. *p < 0.05; **p < 0.01; n.s., not significant; two-tailed.

Given the results of retrograde tracing, possible receptive field changes of V2 VPM neurons after IONC were examined. In anesthetized mice, multiunit activity of V2 VPM neurons was recorded in response to mechanical stimuli throughout the face and body (six and five mice for sham and IONC, respectively; Fig. 6*A–D*). In the sham group, receptive fields were always confined to the V2 region of the contralateral face: 39 V2-responsive sites and three nonresponsive sites of 42 total mapped sites in the V2 VPM (Table 2). These responses were always reliable and have small receptive fields confined to typically one or two whiskers (Fig. 6*C*_(i),(ii)_). However, in the IONC group, receptive fields of V2 VPM neurons were rarely observed on the V2 region of the face (in total, three out of 65 mapped sites); these infrequent V2 responses might have been mediated by the regenerated ION (Waite, 1984; Kis et al., 1999) or direct tension to proximal stump of the ION and/or adjacent region when a heavy von Frey filament was applied to the whisker pad. Importantly, after IONC, receptive fields were observed on the V3 region of the face such as lower jaw and/or also on the body such as neck, chest, and back (in total, nine V3-responsive sites, 11 DCN-region-responsive sites, and 50 nonresponsive sites out of 65 mapped sites; Fig. 6*C*_(iii)_,*D*; Table 2). The nontrigeminal receptive fields (DCN-region-responsive sites) in the V2 VPM were not confined to the ventral most recording sites, which is adjacent to the VPL, but were distributed along the electrode tracks (Fig. 6*C*, red and magenta circles). The ectopic receptive fields were spatially consistent with ectopically labeled projection neurons in the V3 regions of trigeminal nuclei and DCN (Fig. 5). These ectopic responses were not as reliable as normal responses in the sham group, which means repeated stimulations on the ectopic receptive fields do not always fire V2 VPM neurons as those on normal receptive field in the sham group (50–75% and 100% in success rate approximately for the IONC and sham groups, respectively, when similar light tactile stimuli were applied at 0.1 Hz). This observation was very consistent with the fact that there exists a subset of much weaker lemniscal fiber-mediated EPSCs after IONC in the VPM, which may not be strong enough to drive reliable firing of postsynaptic V2 VPM neurons (Takeuchi et al., 2012). The possible functional immaturity of newly invading ectopic fibers could explain a discrepancy between small number of V3- and DCN-region-responsive sites in receptive field recordings and extensive ectopic retrograde labelings in V3 and DCN regions (Figs. 5, 6). In addition, the size of the ectopic receptive fields was much larger (Fig. 6*D*). These results suggest that IONC-induced remodeling of lemniscal fibers, including both PrV2-origin and non-PrV2-origin ones, establishes ectopic sensory pathways from non-V2 peripheral tissue, including the V3 region of the face and the body, to the V2 VPM within one week.

**Table 2.** Receptive fields of V2 VPM neurons one to two-weeks after operations

Operation	RF location	Corresponding brain stem nuclei	Number of recordings	Proportion to total mapped sites (%)
Sham	Whisker(s) or whisker pad	PrV2, V2 SpI	39	92.9
	Nonresponsive	None	3	7.1
IONC	Whisker(s) or whisker pad	PrV2, V2 SpI	3	4.6
	Lower jaw	PrV3, V3 SpI	9	13.8
	Chest	DCN	6	9.2
	Fore paw	DCN	3	4.6
	Hind paw	DCN	1	1.5
	Trunk	DCN	1	1.5
	Tail	DCN	1	1.5
	Nonresponsive	None	50	76.9

Recordings were made from the right V2 VPM. All receptive fields (RFs) were found in the contralateral (left) side of the face and/or body. For the sham group, 42 recording sites were mapped from six mice (six penetrations) in total. For the IONC group, 65 recording sites were mapped from five mice (seven penetrations) in total. Note that in several cases each recording site had multiple RFs such as the lower jaw and the chest. PrV2, maxillary region of the principle trigeminal nucleus; PrV3, mandibular region of the principle trigeminal nucleus; V2 SpI, maxillary region of the interpolar subnucleus of the spinal trigeminal nuclei; V3 SpI, mandibular region of the interpolar subnucleus of the spinal trigeminal nuclei.

To investigate the impact of the IONC-induced lemniscal fiber remodeling on somatotopic sensation, we investigated nociceptive pain behavior by measuring the escape threshold in response to mechanical stimuli applied to the face using the von Frey test (Fig. 6*E*). In the IONC group, the escape threshold in response to mechanical stimuli to the V2 region significantly increased (mostly up to cutoff: 2 g). Note that although normalized escape thresholds of the IONC group tended to decline over the PODs, they are at least 10 times larger than those of the sham group over two weeks after IONC (Fig. 6*E*). The decline of normalized escape thresholds was mediated mainly by increase in the sham group due to growth and partial by decrease in the IONC group possibly due to regeneration of the transected ION. Absolute escape thresholds of the V2 region on POD1 and POD11 were 0.04 ± 0.02 and 0.14 ± 0.18 for the sham group, and 2.00 ± 0.00 and 1.54 ± 0.37 for the IONC group, respectively. This partial recovery in escape thresholds of V2 region might have been due to the same causes for infrequent V2 region receptive fields of V2 VPM neurons described above: ION regeneration and propagations of applied tension to surrounding regions. One to two weeks after IONC, escape thresholds in response to mechanical stimuli to the V3 region (lower jaw) significantly decreased compared with those in the sham group (*p* < 0.001^u^, two-way repeated measures ANOVA, *F*_(1,212)_ = 34.8; *p < 0.05, **p < 0.01, *post hoc* Tukey HSD test; Fig. 6*E*). Decrease in the escape threshold became statistically significant from POD6, which was temporally consistent with that of the lemniscal fiber remodeling (Fig. 2; Takeuchi et al., 2012). These results indicate allodynia-like extraterritorial mechanical hypersensitivity in the mandibular region that spatially corresponds to ectopic receptive fields of V2 VPM neurons after IONC, and these changes were temporally consistent with lemniscal fiber remodeling.

### Long-lasting lemniscal fiber remodeling, reorganization of receptive field, and behavioral changes after IONC

**Figure 7.**
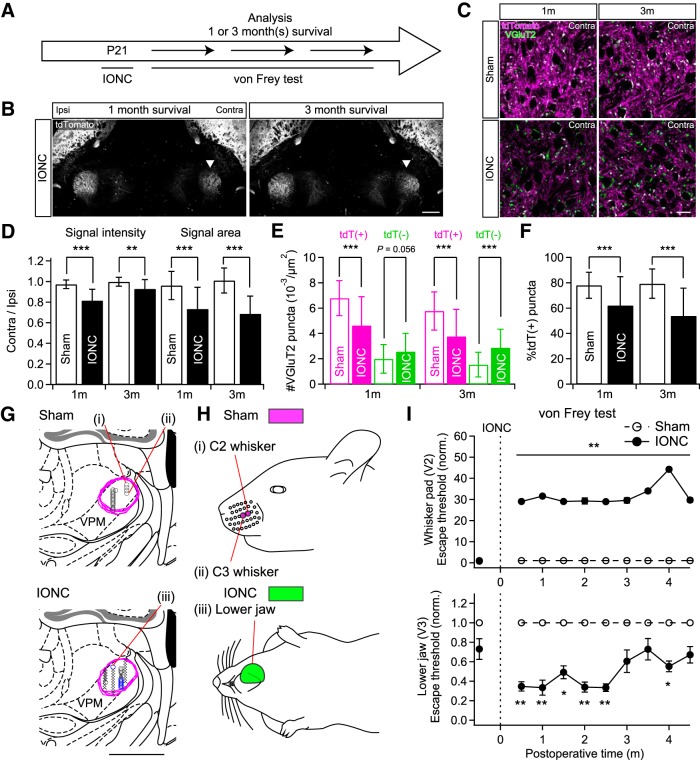
Long-lasting lemniscal fiber remodeling related to ectopic receptive fields of V2 VPM neurons and mechanical hypersensitivities on ectopic receptive fields. ***A***, Experimental schedule. ***B***, tdTomato signals in the V2 VPM of Krox20-Ai14 mice long after IONC. Arrowheads indicate the affected V2 VPM. Scale bar, 500 µm. ***C***, VGluT2-immunostained sections of the V2 VPM. Significant increases of tdT-negative VGluT2 puncta after IONC. Scale bar, 20 µm. ***D***, Ratios of contralateral to ipsilateral tdTomato signals of the V2 VPM in fluorescent intensity and area. Four mice (four images from each mouse) were examined for each group. ***E***, Summary density of VGluT2 puncta in the contralateral V2 VPM. tdT(+) = tdTomato-positive VGluT2; tdT(-) = tdTomato-negative ones. ***F***, IONC decreased proportion of tdT-positive VGluT2 puncta in the contralateral V2 VPM. Four mice (eight images from each mouse) were examined for each group in ***E*** and ***F***. ***G***, Recording sites located by electrical lesion made after receptive field recordings. All recordings made in the V2 VPM were analyzed and reconstructed on the brain atlas of Franklin and Paxinos (2008). Black circles = recording sites of maxillary receptive fields only; blue circles = recording sites of mandibular receptive fields; multiplication signs = nonresponsive sites. Magenta brackets indicate the V2 VPM regions. Roman numerals indicate the recording sites of representative recordings shown in ***H***. Scale bar, 1 mm. ***H***, Representative receptive fields of V2 VPM neurons. Each Roman numeral corresponds to the recording from the site indicated in ***G***. Colored areas on the mouse cartoons indicate receptive fields at the recording sites. Magenta = sham; green = IONC. ***I***, Face-escape thresholds assessed by von Frey filament stimuli on whisker pad and lower jaw before and 0.5–4.5 months after IONC operation on P21. Nerve regeneration was prevented for long-term evaluation (see Materials and Methods). Each value was normalized to that of the sham group. Nine mice were examined for each group. Values are represented as mean ± SD. Statistical significance was tested by two-way repeated measures ANOVA (main effect of IONC; ***D–F***) and two-way repeated measures ANOVA and *post hoc* Tukey HSD test (***I***). *p < 0.05; **p < 0.01; ***p < 0.001; two-tailed.

Next, we investigated the duration of the IONC-induced changes (Fig. 7*A*). One month (1 m) and even three months (3 m) after IONC, tdTomato fluorescence intensity and the occupying area in the contralateral V2 VPM remained significantly reduced [contra/ipsi ratio of signal intensity: 0.82 ± 0.11 and 0.93 ± 0.09 (0.82 ± 0.13 and 0.93 ± 0.09 between animals) for 1 m and 3 m, respectively; contra/ipsi ratio of signal area: 0.73 ± 0.21 and 0.69 ± 0.17 (0.73 ± 0.25 and 0.69 ± 0.27) for 1 m and 3 m; **p < 0.01^v^, ***p < 0.001^v^, main effect of IONC, *F*_(1,24)_ > 8.0 for all; Fig. 7*B*,*D*]. Although there was some recovery between one and three months after IONC in tdTomato fluorescence intensity (**p < 0.01^w^, main effect of survival time, *F*_(1,24)_ = 10.9), there was no recovery in occupying area between one and three months after IONC (*p* = 0.439^x^, *F*_(1,24)_ = 0.6; Fig. 7*B*,*D*). Even three months after IONC, the density of tdT-positive VGluT2 puncta remained lower [105.0 ± 27.6 and 68.7 ± 38.7 (105.0 ± 45.2 and 68.7 ± 62.2 between animals) in 18,212 µm^2^ ROI for sham and IONC, respectively; ***p < 0.001^y^, main effect of IONC, *F*_(1,56)_ = 22.7] and that of tdT-negative ones remained higher [28.0 ± 17.8 and 52.5 ± 26.3 (28.0 ± 39.2 and 52.5 ± 15.3) in 18,212 µm^2^ ROI; ***p < 0.001^z^, *F*_(1,56)_ = 20.6; Fig. 7*C*,*E*]. Consequently, the proportion of tdT-positive puncta remained lower [79.2 ± 11.5% and 53.9 ± 21.8% (79.2 ± 20.4% and 53.9 ± 23.4, ***p < 0.001^aa^, *F*_(1,56)_ = 35.9; Fig. 7*C*,*F*]. Consistent with this long-lasting and large-scale lemniscal fiber remodeling, ectopic receptive fields were found more than one month after IONC (*n* = 3 mice for each group). Like the results in Figure 6, receptive fields of V2 VPM neurons typically included the V3 region of the face (sham: 20 V2-responsive sites, one nonresponsive site out of 21 total mapped sites; IONC: seven V2-responsive sites, eight V3-responsive sites, 38 nonresponsive sites out of 52 total mapped sites; Fig. 7*G*,*H*; Table 3). The size of such ectopic receptive fields was much larger than that in the sham group. Furthermore, more than three months after IONC, a lowered escape threshold in response to mechanical stimuli on the V3 region still existed (*p* < 0.001^bb^, two-way repeated measures ANOVA, *F*_(1,50)_ = 51.2; *p < 0.05, **p < 0.01, *post hoc* Tukey HSD test; Fig. 7*I*). These results indicate that the IONC-induced lemniscal fiber remodeling in the V2 VPM, ectopic receptive fields of V2 VPM neurons, and ectopic mechanical hypersensitivity are all long-lasting with temporal consistency among them.

**Table 3.** Receptive fields of V2 VPM neurons one to three months after operations

Operation	RF location	Corresponding brain stem nuclei	Number of recordings	Proportion to total mapped sites (%)
Sham	Whisker(s) or whisker pad	PrV2, V2 SpI	20	95.2
	Nonresponsive	None	1	4.8
IONC	Whisker(s) or whisker pad	PrV2, V2 SpI	7	13.5
	Lower jaw	PrV3, V3 SpI	8	15.4
	Nonresponsive	None	38	73.1

Recordings were made from the right V2 VPM. All receptive fields (RFs) were found in the contralateral (left) side of the face and/or body. For the sham group, 21 recording sites were mapped from three mice (three penetrations) in total. For the IONC group, 52 recording sites were mapped from three mice (five penetrations) in total. Note that in several cases each recording site had multiple RFs such as the lower jaw and the whisker pad. PrV2, maxillary region of the principle trigeminal nucleus; PrV3, mandibular region of the principle trigeminal nucleus; V2 SpI, maxillary region of the interpolar subnucleus of the spinal trigeminal nuclei; V3 SpI, mandibular region of the interpolar subnucleus of the spinal trigeminal nuclei.

### IONC induced lemniscal fiber remodeling in the adult

**Figure 8.**
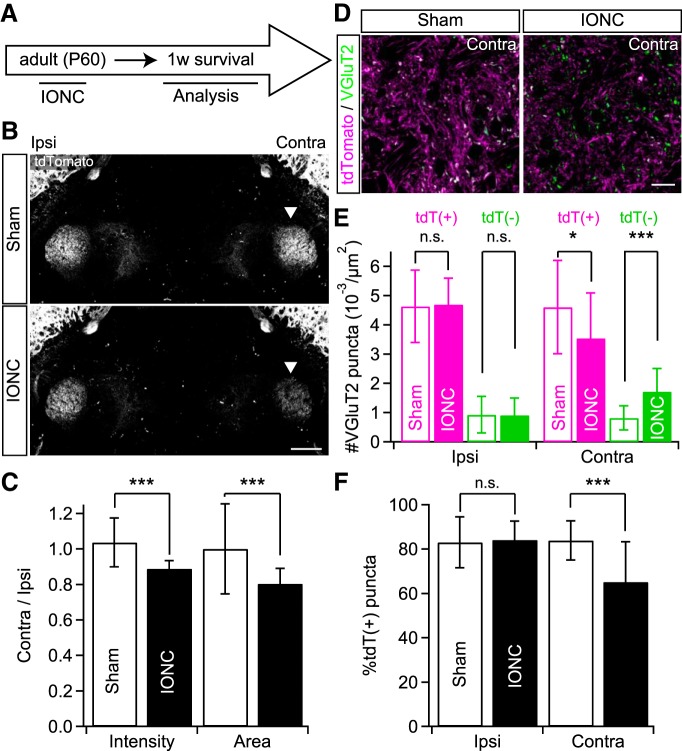
No critical window of IONC-induced lemniscal fiber remodeling. ***A***, IONC operation was conducted on adult Krox20-Ai14 mice. ***B***, tdTomato signals in the V2 VPM of Krox20-Ai14 mice IONC operated in adult. Arrowheads indicate the affected thalamus. Scale bar, 500 µm. ***C***, Ratios of contralateral to ipsilateral tdTomato signals of the V2 VPM in fluorescent intensity and area. Sixteen images from five mice were examined for each group. ***D***, VGluT2-immunostained sections of the contralateral V2 VPM in Krox20-Ai14 mice IONC operated in adult. Scale bar, 20 µm. ***E***, Summary density of VGluT2 puncta in the V2 VPM. tdT(+) = tdTomato-positive VGluT2; tdT(-) = tdTomato-negative ones. ***F***, Summary proportion of tdT-positive VGluT2 in the V2 VPM. Three mice (eight images from each mouse) were examined for each group in ***E*** and ***F***. Values are represented as mean ± SD. Statistical significance was tested by two-way repeated measures ANOVA (main effect of IONC; ***C***, ***E***, and ***F***). *p < 0.05; ***p < 0.001; n.s., not significant; two-tailed.

We also investigated whether IONC in adult animals induced the lemniscal fiber remodeling in the V2 VPM (Fig. 8*A*). One week after IONC in adults (P60), we macroscopically observed significantly decreased tdTomato fluorescence intensity and occupying area in the contralateral V2 VPM [contra/ipsi ratio: 0.89 ± 0.05 and 0.80 ± 0.09 (0.89 ± 0.05 and 0.80 ± 0.04 between animals) for intensity and area, ***p < 0.001^cc^, main effect of IONC, *F*_(1,26)_ > 14.5 for both; Fig. 8*B*,*C*]. After the adult IONC procedure, the density of tdT-positive VGluT2 puncta significantly decreased [83.8 ± 29.0 and 64.5 ± 28.1 (83.8 ± 33.1 and 64.5 ± 21.3 between animals) in 18,212 µm^2^ ROI for sham and IONC, respectively; *p = 0.024^dd^, main effect of IONC, *F*_(1,42)_ = 5.5], whereas that of tdT-negative ones increased [14.8 ± 7.5 and 31.3 ± 14.4 (14.8 ± 12.7 and 31.3 ± 4.0) in 18,212 µm^2^ ROI; ***p < 0.001^ee^, *F*_(1,42)_ = 24.1] in the contralateral V2 VPM (Fig. 8*D*,*E*). The total density of VGluT2 puncta did not differ between sham and IONC groups [98.6 ± 28.8 and 95.8 ± 20.0 (98.6 ± 40.3 and 95.8 ± 17.8) in 18,212 µm^2^ ROI; *p* = 0.686^ff^, *F*_(1,42)_ = 0.166]. Accordingly, the percentage of tdT-positive VGluT2 puncta in the contralateral V2 VPM significantly decreased after IONC even in adults [83.9 ± 8.8% and 65.2 ± 18.2% (83.9 ± 12.6% and 65.2 ± 6.9%); ***p < 0.001^gg^, *F*_(1,42)_ = 19.6; Fig. 8*D*,*F*]. Thus, it is likely that the IONC-induced lemniscal fiber remodeling has no critical time window.

## Discussion

### Neural circuitry mechanisms underlying large-scale somatotopic reorganization in the thalamus after peripheral sensory nerve injury

Using a Krox20-Ai14 transgenic mouse line combined with VGluT2 immunohistochemistry, we successfully distinguished somatotopic information for remodeled lemniscal fiber at single-bouton resolution after IONC. We found that the number of EPSC steps and percentage of non-PrV2-origin lemniscal fiber terminals in the V2 VPM were significantly correlated (Fig. 2). The correlation suggests that multiple non-PrV2-origin lemniscal fibers converged onto a V2 VPM neuron after IONC. Whisker deprivation, on the other hand, induced neither multiple lemniscal fiber innervations onto a V2 VPM neuron (Takeuchi et al., 2012) nor invasion of ectopic inputs (Fig. 2), confirming that these changes were induced by nerve injury itself, but not by sensory deprivation. Furthermore, the results shown in Figure 5 demonstrated clearly that the lemniscal fiber remodeling is associated with new projections of ectopic afferent inputs from large-scale uninjured origins, including PrV3, V3 SpI, and DCN to the V2 VPM. It is likely that sprouting collaterals of V3 VPM-targeted and/or VPL-targeted lemniscal fibers originating from these uninjured brainstem nuclei constitutes a neural circuitry mechanism underlying the somatotopic reorganization after IONC. This speculation is quite reasonable because the V3 VPM and VPL are located in regions adjacent to the V2 VPM (Franklin and Paxinos, 2008). Taken together, the present study provides clear evidence of a neural circuitry mechanism underlying large-scale somatotopic reorganization in the thalamus after peripheral sensory nerve injury.

### Spatial and temporal consistency among lemniscal fiber remodeling, reorganization of receptive fields, and abnormal ectopic sensation after IONC

The somatotopic reorganization underlying anatomic remodeling of lemniscal fibers could be further expanded to changes in receptive fields of V2 VPM neurons and behavioral level in terms of spatial and temporal consistency. Spatially, ectopic non-PrV2-origin lemniscal fibers, ectopic receptive fields, and extraterritorial mechanical hypersensitivity all occurred on the mandibular and DCN regions (Figs. 5, 6). Temporally, we confirmed that all changes in our experiments at different hierarchical levels, including multiple lemniscal fiber innervation onto a V2 VPM neuron (Takeuchi et al., 2012), remodeling of lemniscal fiber synapses in the V2 VPM (Fig. 5), changes of PrV2-origin lemniscal fiber terminal morphology (Fig. 4), appearance of ectopic receptive fields of V2 VPM neurons (Fig. 6) and extraterritorial mechanical hypersensitivity on the mandibular region (Fig. 6), concomitantly developed within one week after IONC. These changes followed a similar time course over three months (Fig. 7), which is clearly different from the time course of unmasking of preexisting but latent neural pathways (unmasking emerges within a few minutes and lasts several hours after lidocaine-mediated deafferentation; Nicolelis et al., 1993). A similar time course as the IONC-induced change in receptive fields of V2 VPM neurons has also been reported in the somatosensory cortex after sciatic nerve transection in rats (Wall and Cusick, 1984; Pearson et al., 1999) and median nerve transection in adult monkeys (Merzenich et al., 1983). Thus, the somatotopic reorganization in the thalamus induced by the peripheral sensory nerve injury may in turn influence large-scale receptive field changes in the somatosensory cortex observed years after dorsal root lesion (Pons et al., 1991; Jones, 2000). The consistency among our neuroanatomical and behavioral results strongly suggests that the thalamic circuit remodeling mediating somatotopic reorganization, changes in thalamic receptive fields, and mechanical hypersensitivity occur within a series of processes that are interdependent.

In addition to the general spatial and temporal consistency on our data leading to above conclusion, it is worthwhile to note that there were several changes of our data along postoperative period. First, ectopic receptive fields mediated by DCN such as what evoked by chest stimulation were present until two weeks after IONC but absent over one month after IONC (Figs. 6, 7; Tables 2, 3). Therefore, after IONC, ectopic DCN-origin lemniscal fibers seem to invade the deafferented V2 VPM once, and they would then be eliminated by one month after IONC. Second, there was significant recovery in tdTomato fluorescence intensity of PrV2-origin lemniscal fibers in the V2 VPM between one month and three months after IONC although it remained still statistically decreased compared with the survival-period-matched sham groups (Fig. 7*D*). It means restrengthening of once weakened original whisker-sensory pathway. These changes could be considered as the subsequent second reorganization after the initial large-scale somatotopic reorganization both mediated by lemniscal fiber remodeling. In the primary somatosensory cortex of nonhuman primates deafferented by chronic dorsal column lesion, reactivation of the original receptive fields occurs over initial two months (Jain et al., 1997), which is very similar to our results (Fig. 7). The reactivation is followed by large-scale somatotopic reorganization by invasion of ectopic face representation in both the primary sensory cortex and the somatosensory thalamus over six months after the deafferentation (Jain et al., 1997; Jain et al., 2008). Thus, pursuits on further long-term reorganization in our model and comparison with these previous nonhuman primate studies would provide fruitful insights of somatotopic reorganization in future studies.

### Injury-induced remodeling of the somatotopic pathway in the adult

Because the IONC-induced lemniscal fiber reorganization in the V2 VPM was maintained into adulthood (Fig. 7) and was induced in both juvenile and adult mice (Fig. 8), we conclude that there is not a critical time window for this injury-induced lemniscal fiber remodeling. This is consistent with the results in previous studies showing that injury of the primary sensory neurons by amputation, dorsal root lesion, or dorsal column lesion induces thalamic anatomic and functional changes in adult human and nonhuman primates: withdrawal of lemniscal axons (Graziano and Jones, 2009), and large-scale somatotopic reorganization (Davis et al., 1998; Jones and Pons, 1998; Florence et al., 2000; Jain et al., 2008). In the present study, we demonstrated that IONC in the adult resulted in anatomic remodeling including the invasion of ectopic lemniscal fiber terminals (Fig. 8). Thus, it is expected that IONC in the adult would also cause large-scale receptive field reorganization and extraterritorial mechanical hypersensitivity (e.g., data shown in Figures 6 and 7).

In the visual thalamus, there is a critical time window for sensory deprivation-induced retinogeniculate fiber remodeling. Visual deprivation after P20, induces the remodeling onto a relay neuron in the visual thalamus of the mouse (Hooks and Chen, 2008). In the VPM, whisker deprivation within a critical time window (P12–P13) disrupts developmental synapse elimination (Wang and Zhang, 2008; Zhang et al., 2013). Thus, we conclude that the IONC-induced lemniscal fiber remodeling demonstrated in our study should rely on other mechanisms different from these developmental processes in the thalamic circuits.

### Functional implications of lemniscal fiber remodeling in the somatosensory thalamus after peripheral sensory nerve injury

We observed allodynia-like mechanical hypersensitivity on the uninjured mandibular region after IONC. Similar extraterritorial pain has been reported in cases of the inferior alveolar nerve transection and cervical spinal nerve injury in patients and animal models, which cause abnormal orofacial pain in the uninjured face region (Kobayashi et al., 2011; Renton et al., 2012; Tseng et al., 2012; Okada-Ogawa et al., 2015). It has been proposed that changes of excitability in the trigeminal ganglion and SpC possibly contribute to this extraterritorial orofacial pain (Kobayashi et al., 2011; Takeda et al., 2011; Okada-Ogawa et al., 2015). Currently, it remains unclear how allodynia-like mechanical hypersensitivity develops after IONC. Intraganglionic and intrabrainstem functional reorganization might contribute to the abnormal sensation as these previous studies have proposed. Increased projections from the SpC to the V2 VPM after IONC might also have some contribution (Fig. 5). In the present study, both thalamic somatotopic reorganization and emergence of extraterritorial pain behavior occurred with spatiotemporal consistency, suggesting that somatotopic reorganization at the thalamic level may also be involved in extraterritorial orofacial pain. On the other hand, it is widely considered that somatotopic reorganization is an essential element for phantom referred sensation that can often be induced by tactile stimulation on particular body parts in the amputees (e.g., tactile stimulation on the face induces abnormal sensations on the lost arm/fingers; Ramachandran and Hirstein, 1998). It is thought that peripheral nerve injury causes a contamination of ectopic afferent information in the somatosensory afferent pathway; the abnormal mismatch between incoming and outgoing somatotopic information in the CNS results in distorting perception of own body images, leading to the phantom sensation (Kew et al., 1997; Davis et al., 1998; Grüsser et al., 2004; Flor et al., 2006). In the somatosensory thalamus, microstimulation of the VPL in the amputees whose thalamic receptive fields are largely reorganized can induce phantom sensations (Davis et al., 1998). This result supports the idea that thalamic receptive field of an intact body part expands into the missing limb region of the thalamus after the loss of limb inputs (Davis et al., 1998) and is consistent with axonal sprouting of afferent fibers in the somatosensory thalamus including our own results in the present study (Florence and Kaas, 1995; Sengelaub et al., 1997; Florence et al., 1998; Jain et al., 2000). Taken together, we propose that remodeling of neural circuit at thalamic levels could underlie the emergence of abnormal ectopic sensations such as extraterritorial orofacial pain and phantom referred sensation.

## Synthesis

Reviewing Editor: Karen Davis, Krembil Research Institute and University of Toronto Decisions are customarily a result of the Reviewing Editor and the peer reviewers coming together and discussing their recommendations until a consensus is reached. When revisions are invited, a fact-based synthesis statement explaining their decision and outlining what is needed to prepare a revision will be listed below. The following reviewer(s) agreed to reveal their identity: Preston Garraghty, Neeraj Jain.

This study uses a transgenic mouse line to visualize afferent fiber remodeling in the somatosensory thalamus after nerve injury. Specifically, they show that after this injury, in the VPM there is a decrease in terminal spread of whisker inputs from the principal trigeminal nucleus. There is an increase in synaptic inputs from non-whisker afferents from principal and spinal trigeminal nuclei, and novel inputs from the cuneate nucleus and gracile nuclei are also seen. These changes were not seen after 7 - 11 days of whisker deprivation, and thus are not seen after short sensory deprivation. The results are supported by electrophysiological and behavioural data. Finally, they show that a decrease in whisker inputs related puncta and increase in non-whisker related puncta is also seen after ION cut in 60 day old adult mice, concluding that this transneuronal change can bee seen even in adult animals. The data is exhaustive, well presented and discussed, and provide evidence for one of the many possible mechanisms of brain plasticity.

The following major modifications are requested to improve the paper:

1. Emergence of inputs from cuneate and gracile nuclei (Fig. 5) after ION cut is one of the intriguing and interesting finding of the this work. The number of receptive fields in VPM of non-trigeminal origin are few (Table 1), although the retrograde labeling in DCN is extensive (Fig 5C). This interesting observation can be further elaborated. Were these few receptive fields at the ventral most recording sites, i.e. adjacent to VPL? Their location along the electrode track can be illustrated.

Authors have managed the technically challenging task of injecting a tracer that is restricted to a tiny sub-region of mouse VPM. It would be nice to see few illustrative injection sites from normal and nerve-cut mice. In legend to Fig. 5A it is mentioned that both sham and ION cut animals are shown, but I see only one figure.

2. Touching a mouse, which is held in experimenters hand, with increasing force using von Frey hair, and interpreting 'aversive behavior' of swiping at the filament with the forepaw as an indicator of allodynia appears to be a little bit of stretch. Is it just tactile sensitivity?

Also the escape threshold for touching whisker pad also seems to show a trend of decreasing to pre-ION cut level (Fig. 6E, left). This observation needs to be presented and discussed.

3. It would be useful of the authors could show the data presented on page 18, line 395 as jitter plots similar to that shown in figure 4C2. This will help the reader better judge the results since the SD is high.

In addition, please address the following minor issues:

Line 107: add 'mice' at the end of the sentence.

Line 369: Sentence needs to be reworked.

Line 371: Change underling to underlying; move Graziano reference to the end of the sentence. The fragment 'a marker of lemniscal fiber terminals' can be a separate sentence for better readability.

Line 494: Change 'on' to 'of'
